# Pre‐Registration Assessment of Bone‐Filling Products

**DOI:** 10.1111/os.12499

**Published:** 2019-09-06

**Authors:** Shuo Pan, Bin Liu, Yue Min, Jia‐yi Sun, Bao Zhai, Xiao‐heng Guo

**Affiliations:** ^1^ Center for Medical Device Evaluation NMPA Beijing China

**Keywords:** Bone‐filling material product, Experimental operations, Technical indices

## Abstract

The use of bone‐filling material to repair bone defects and fix implanted bone grafts is a developing area in medicine. Investigators can evaluate bone‐filling materials through use of several indices to make comparisons and to determine suitability for application in humans^1^. However, it is quite difficult to transform their discovery into practical use, because the viability of the studied material might require examination of all aspects of properties. In addition, for a material to become a product, a complete procedure involving a declaration, registration, and approval is necessary. This article introduces the technical indices that the investigators and reporters should provide in their declaration and registration data to meet the relevant standards in China. The indices include physical and chemical properties, biocompatibility, biosecurity, pre‐clinical animal model tests, sterilization and disinfection, product duration, and packaging. Full consideration of all possible indices is crucial to realize the transformation from a designed product to a commercial medical device, which requires effective interaction between clinicians and engineers.

## Introduction

For decades, orthopaedic studies have been investigating appropriate materials to fill defects in human bone. Use of bone‐filling material for repairing bone defects and fixing implanted bone grafts is a developing area of research. Researchers seek out special materials that not only have mechanical properties that resemble those of bone, but also have excellent biocompatibility to enable bone fusion.^1^ In addition, there are many other indices that should be considered to assess the viability of the material. A few materials have been proved to exhibit good properties to fill bone defects, such as demineralized bone matrix (DBM)^2^, tricalcium phosphate (TCP)^3^, and calcium sulfate (CS)^4^.

Despite a material having promising medical applications and excellent properties for bone filling it is difficult to reach the commercial stage. The main obstacles are the investigations into and the assessment of the viability of the material. This requires interaction between clinicians and engineers to transform the designed product into a commercial medical device. Certainly, the confirmatory experiments should be conducted according to the relevant national standards and announcements.

*GB/T 16886* Standard Series and *ISO 10993* Standard Series list biological assessment methods for medical devices, providing an outline for structural procedures in biosecurity assessment. They also illustrate assessment methods for medical devices in regard to physical and chemical properties, biological properties, and risk assessments. *Opinions on Deepening the Reform of the Evaluation and Approval Systems and Encouraging Innovation on Drugs and Medical Devices*, issued by the General Office of the Communist Party of China (CPC) Central Committee and the General Office of the State Council, suggests essential strategies for clinical testing and administration of medical devices. *The Announcement on Formations of Resource Requirements and Approval Demonstrative Files of Registering and Declaring Medical Devices* (shortened as *The Announcement*) describes the procedures for approving and assessing organizations. *The Investigation and Instruction on the registration of CaP/CaSi‐based bone filling materials* (shortened as *The Investigation*) lists the data that should be submitted during the registration process, and the relevant experiments that should be conducted before registration to demonstrate the viability in regards to every tested property.

This article aims to: (i) provide technical support for bone‐filling material registration and declaration; and (ii) discuss the potential experimental procedures to carry out for examination of product viability, to reasonably and efficaciously accomplish the declaration and registration of bone‐filling materials.

## General Information

Generally, the reporter should describe the chemical components, the processing methods, the sterilization types, and the expected uses. The source of control samples (including positive and sensitive control) used in the experiments and the reasons for applying them should be stated. The declared product should be set as the experimental group, and the same or similar type of listed products as the positive control group. A negative control group may also be required for conducting a control test of relevant properties.

For each study and assessment report of the relevant property index, the reporter should illustrate in detail whether the product requires the study to be based on this index, and then state the reasons. For the property index studied, the experimental methods, results, discussions, and conclusions should be provided. For any quantitative or qualitative tests, the testing results should meet the requirements for data or criteria as stated in the national standards. For the properties indices that are not mentioned in the national standards but possibly required, the reporter should not only illustrate the experimental procedures, but also state the reasons for performing the experiments as well as whether the results meet the requirements of relevant articles or international standards quantitatively or qualitatively.

## Physical and Chemical Properties

The physical and chemical properties assessed for the product shall include, but not be restricted to, chemical composition, mechanical properties, density, porosity, and surficial features.

### *Chemical Composition*

For every single product, the reporter should indicate its chemical composition and the proportion of each component^5^. For mixed solid–liquid phase products (such as paste and putty), the ratio of weight between two phases should also be indicated. The reporter should discuss briefly whether there are possible chemical reactions between the product and the human body when the product directly or indirectly contacts body parts (such as skin, blood, and tissues), and what they are. For a chemical component that has already been proved to be toxicologically hazardous, appropriate experiments should be designed and the experimental process for *in vitro* toxicity should be recorded.

### *Mechanical Properties*

When detecting its mechanical properties, the product should be closely connected with its material type and shape. For a solid product, the investigator should consider its compressive strength^6^. Relevant computer software could be applied to determine the stress distribution and the ability of the product to withstand a certain value of stress. In addition, for paste and putty products, more attention should be paid to the diametral tensile strength, the compressive strength, the consistency, and the viscosity. The consistency, viscosity, and solid–liquid weight ratio might impact its injectability.

### *Density and Porosity*

As for the product with the same type of model, its density could be derived by the weight over volume^7^. The porosity defines the volume of pores over the whole volume, and its equation gives:

$$P=\frac{V_{0}-V}{V_{0}}\times 100\%=\left(1-\frac{\rho_{0}}{\rho }\right)\times 100\%,$$

where *V*_0_ is the volume of the original product, *V* is the volume excluding pores, and *ρ*_0_ is the original density of the material. Each parameter could be measured several times and applied as the average. The results could be compared with the control sample product and natural bone product (containing cortical and cancellous bone parts).

### *Surficial Features*

For block‐shaped solid bone‐filling products, the surface roughness could be used to indicate its surficial feature^8^. The surficial roughness could be obtained by zooming into the product surface using an optical microscope and measuring and calculating relevant parameters. The Latter could, to some extent, characterize the wear resistance of the product, which might also be related to its hardness, mechanical strength, environmental temperature. Surface roughness should also be assessed during the pre‐clinical animal model tests, discussed below. For mixed solid–liquid phase products, surface roughness can only be investigated through pre‐clinical animal model testing, as discussed below.

### *Safety Indices*

According to *The Announcement*, the safety indices for the material should include electrical safety, electromagnetic compatibility, and radiation safety^9^. These specific considerations are not relevant for most ceramic products or for ceramic components in products^10^. However, for products containing organic macro‐molecular components, the adverse effects of electricity and radiation should be taken into consideration by the investigator. The reporter should explain whether the safety indices tests should proceed for the product according to appropriate standards in the literature and provide detailed experimental procedures.

### *Other Properties*

Other physical and chemical properties that might be detected in the experimental work include, but are not restricted to, crystallinity (by XRD)^11^, crystal size and distribution, hydrophobicity or hydrophilicity, and hygroscopicity^12^. For products containing ceramic materials, study of trace analysis and micro‐morphology might be required. For products containing organic macro‐molecular materials, the investigator might further examine, for example, the chemical bond configuration, the chemical structure, the molecular distribution, and impurities.

## Biocompatibility

Biocompatibility studies the properties of a product when it makes contact with the biological environment. The factors that should be considered include, but are not restrict to, cellular growth and proliferation, cellular adherence, *in vitro* cellular toxicity, and degradation^13^. It should be noted that some of the *in vitro* tests might be processed in a excessive strict condition compared to the practical conditions^14^. In addition, *in vitro* test results sometimes do not represent the real results. Hence it is crucial to implement pre‐clinical animal model tests.

### *Cellular Growth and Proliferation*

This subsection investigates the ability of the bone‐related cells to grow and proliferate in the environment of the product as a scaffold^15^. A certain amount of the studied product is applied in a culture medium with cultured osteoblasts and other appropriate conditions, and this is set as the experimental group. A commercially available bone‐filling product (such as the product from Wright Medical), with the same conditions as in the experimental group to culture the osteoblasts, is used for the positive control group^16^, ^17^. The osteoblasts and the same conditions in the medium without any product are set as the negative control group. The number of mediums containing the product (or not) should be set up comparably. The product samples are periodically taken out, sectioned and dyed, then the cell growth and proliferation are observed through an optical microscope. The number of cells per unit area is estimated by meshing the microscopic images, and the total amount of cells in the section is extrapolated. The relation between time and cell amount can be presented using a curve graph, indicating the cell proliferation ability in the related product.

### *Cellular Adherence*

Cellular adherence is not only a basic condition for stabilizing the tissue structure, but a regulatory factor for cell dynamics and cellular functions. It attaches great importance to the cellular proliferation and differentiation^18^. Cellular adherence can be divided into cell‐to‐cell adherence and cell‐to‐matrix adherence, in which the latter is related to the studied product. Common methods include mechanical process, radionuclide, and cell separating tests. The reporter should illustrate the applied experimental method with the reason, state the experimental process, and record the cell behaviors quantitatively. Finally, by comparing the results for groups, the reporter should conclude whether cell adherence is effective with the studied product.

### *Cellular Toxicity*

Before *in vitro* cellular toxicity testing, the reporter could indicate the components in the product that are possibly toxic, and that might influence cellular activity, based on the literature^15^. The experimental work could be processed with the experimental group and control groups. The product samples are periodically taken out, sectioned, and dyed, and the cellular morphology is observed through an optical microscope. According to GB/T 16886.5–2017, the *in vitro* cellular toxicity can be determined using either quantitative or qualitative methods. The quantitative method provides the ratio of deformed and damaged cells among all per unit areas, and extrapolates the cell activity in the related product. The qualitative method estimates the ratio of deformed and damaged cells and subjectively divides the results into gradient levels.

### *Degradation*

The degradation process for bone‐filling materials can be divided into two types: the ionization of the ceramic component in the product and the absorbance of ions by related cells and tissues (such as the ionization of β‐TCP into Ca^2+^ and PO_4_^3−^); and the decomposition of organic macro‐molecules into small particulates (physically) or micro‐molecules (chemically) and the absorbance by related cells and tissues (such as the absorbance of bone morphogenetic protein in DBM)^19^.

The reporter should indicate whether the product components have potential degradation properties according to relevant literature, and discuss the necessity of the degradation test. According to GB/T 16886.9–2017^20^, the degradation test could be neglected if the clinical experiments and relevant statistics of the studied product have been confirmed to be similar as the degradation properties and outcomes of a product with similar contributing components in the market. For a product that has components with potential degradation properties but without clinical and statistical confirmation, the experimental scheme should be meticulously designed to assess the property. Before the experiment, the characteristics of the product, such as chemical components, chemical properties, biological properties, surface morphology, and expected applications should be determined, together with possible degradation procedures and outcomes. The experimental scheme could focus on several aspects:

- Environmental conditions during the degradation process, including the illumination, temperature, and catalysts in the solution such as enzymes.
- The composition of degradation outcomes, which could possibly be determined by XRD, EDX, DSC, and FTIR. The outcome components should be compared with the original material for discussion of the mechanism.
- The characterization and properties of the degradation outcomes. The degradation outcomes might include water soluble substance such as ions or micro‐molecular organics, or water‐insoluble particulates. As for the water‐soluble outcomes, the concentration should be tested with appropriate methods. For insoluble particulates, the investigator should determine their physical characteristics, such as their morphology, dimensions, and surface area.
- The quantitative study of the degradation outcomes. For example, a product is crushed into powder and further diluted into a given strong‐polarity solution (or a simulated body fluid solution) and filtered in 120 ± 1 h. The difference between the original sample weight and the residual weight of the particulate is then calculated to give the weight of the dissolved sample.

### *Biological Safety*

For products with biosecurity risk (such as allograft, xenograft, or products containing bioactive substances), relevant data demonstrating their biosecurity should be provided^10^. These include:

- Reports demonstrating the acquisition, processing, storage, and testing and treatment process of the product.
- The sources (including details of donators) or the inactivation process of virus, pathogen and substance with immune stimulation (the reporter should provide detailed experimental procedures).
- A brief summary of technical verification.

## Pre‐clinical Animal Model Test

The pre‐clinical test is necessary before the product is applied in the market. The test subjects should be common, healthy, adult mammals (such as the New Zealand white rabbit)^21^. The types of bone should be selected based on the actual situation, where the cranium, mandible, femur, and tibia are the bones most commonly used. The experimental results should cover the efficacy of the product, advantages and disadvantages, and potential risks of implantation. The indices should include, but not be restricted to, the bone graft fusion rate, the post‐implantation local reactions, stimulation and skin allergy, general toxicity, toxicokinetic of the degradation product, and leachable substance.

### *Bone Graft Fusion Rate*

The investigation reveals that the bone graft fusion rate is a significant index to assess bone‐filling materials in pre‐clinical animal model tests, and qualitatively to assess the situation of fusion degree between the original bone and the applied product^22^. The experimental design should cover the steps of the process in detail, including the selection of animals, anesthesia, acquisition of the related bone parts, sectioning, dyeing, and microscopic observation.

### *Post‐implantation Local Reactions*

This part of the experiment should be conducted according to GB/T 16886.6–2015. The investigator should determine the position of the bone graft implantation and select appropriate animal species. The reporter should assure the least number of animals, periodic group division, and experimental conditions in each group. The local reaction results of the product could be observed by the naked eye and microscopy, and compared with the sample in the control group. Moreover, the reaction degree could be analyzed using a quantitative marking system and a half‐quantitative marking system, the indices of which can be derived from, for instance, the cellular morphology, new vascular formation ability, fibrosis, and fat immersion^18^.

### *Stimulation and Skin Allergy*

This index assesses the possible hazards of the released chemical component from a bone‐filling product when it contacts a body part^23^. First, the investigator should fully consider the material type, component and possible adverse effect induced by the implanting process. Second, the investigator should establish, for instance, the appropriate animal species, the number of animals in each group, and periodic group division. Finally, after the amount and frequency of the product dose to be injected into the relevant body part are accordingly determined, the experiment can proceed. The data can be quantified by the degree of hydro‐derma and formation of erythema. The objective degree of stimulation and skin allergy could be assessed by applying a related marking system and analyzed with results and discussions.

### *General Toxicity*

Because various components from products might induce different toxic reactions during the pre‐clinical tests, the symptoms could be divided into acute, sub‐acute, sub‐chronic, and chronic, according to the degree of reactions. The study should determine the amount and frequency of the product dose to be injected and take into consideration that the dose amount should be related to the physical degradation ability of the product^24^. The experiment could be processed after the minimum number of animals in each group is ascertained. The items to be observed and assessed include weight change, clinical observation, clinical pathology, macro‐pathology, weight of organs, and histopathology. The reporter should summarize and analyze the results for each item.

### *Toxicokinetic of the Degradation Outcome and Leachable Substance*

This index is used to study absorption, distribution, metabolism, and excretion of the degradation outcomes after the implantation of bone‐filling material devices. The study should examine related dynamic parameters, including the absorption rate, the volume of distribution, half‐life, and clearance^24^. The following information should be included in the report:

- The animal strain, source, age, gender, and environmental and food conditions.
- The experimental substance and sample, purity, stability, composition, and food intake.
- Experimental conditions such as food intake methods.
- Testing, extracting, detecting and confirming methods.
- The material recovery rate.
- The graph plotting of the results on each time point.
- The statement of corresponding quality standard and laboratory quality control criteria.
- Results, discussions, and explanations.

### *Surface Roughness and Wear Resistance*

As mentioned above, the surface roughness of the material could be compared between the results before and after implantation. The change in roughness among all experimental and control groups could also be compared to examine the effect of various products. Because it might be affected by the mechanical properties of the product, the wear resistance behavior could be investigated during the pre‐clinical animal tests. The weight loss of the product could be measured by weighting the product before and after the pre‐clinical tests and calculating the difference. The wear resistance results could be presented as a detailed report^25^.

### *Conclusion*

The experimental assessment before declaration and registration attaches great importance to the properties and behavior of the bone‐filling product. The viability of the product should be fully examined and the requirements listed in the related standards should be satisfied. With the pre‐registration indices qualified, the interaction between clinicians and engineers could be more effective in the transformation of a bone‐filling material to a useful medical device in the market.
